# Assessing the Impact of Language Access Regulations on the Provision of Pharmacy Services

**DOI:** 10.1007/s11524-018-0240-z

**Published:** 2018-04-03

**Authors:** Linda Weiss, Maya Scherer, Tongtan Chantarat, Theo Oshiro, Patrick Padgen, Jose Pagan, Peri Rosenfeld, H. Shonna Yin

**Affiliations:** 1 New York Academy of Medicine, New York, NY USA; 2 Minnesota School of Public Health, Minneapolis, MN USA; 3 Make the Road New York, New York, NY USA; 4 Department of Public Health Policy and Management, College of Global Public Health, New York, NY USA; 5 Leonard Davis Institute of Health Economics, University of Pennsylvania, Philadelphia, PA USA; 6 NYU Langone Health, Departments of Nursing, New York, NY USA; 7 Departments of Pediatrics and Population Health, NYU School of Medicine, New York, NY USA

**Keywords:** Language access services, Immigrants, Medication adherence, Prescription medications, Pharmacies, Health policy

## Abstract

Approximately 25 million people in the United States are limited English proficient (LEP). Appropriate language services can improve care for LEP individuals, and health care facilities receiving federal funds are required to provide such services. Recognizing the risk of inadequate comprehension of prescription medication instructions, between 2008 and 2012, New York City and State passed a series of regulations that require chain pharmacies to provide translated prescription labels and other language services to LEP patients. We surveyed pharmacists before (2006) and after (2015) implementation of the regulations to assess their impact in chain pharmacies. Our findings demonstrate a significant improvement in capacity of chains to assist LEP patients. A higher proportion of chain pharmacies surveyed in 2015 reported printing translated labels, access and use of telephone interpreter services, multilingual signage, and documentation of language needs in patient records. These findings illustrate the potential impact of policy changes on institutional practices that impact large and vulnerable portions of the population.

## Introduction

Approximately 25 million people in the United States (US) are limited English proficient (LEP) [1]. Compared to English proficient persons, those who are LEP are more likely to have reduced access to care, have a limited comprehension of health information [2], report lower levels of satisfaction with health care services [3, 4], and may face higher medical costs due to unnecessary diagnostic testing [3]. Appropriate language services can improve care for LEP individuals [3, 4], and health care facilities receiving federal funds, including payments through the Medicaid and Medicare programs, are required—according to Title VI of the Civil Rights Act of 1964 [5, 6]—to provide such services [7].

Although language services are increasingly available in many health care settings [8–10], several studies have demonstrated inadequate provision of language services in pharmacies. In research conducted in New York City (NYC) in 2006, we found that of pharmacies reporting that they had LEP patients on a daily basis, just 39% reported providing translated prescription medication labels daily and 23% never translated labels. Compared to independent pharmacies, chains were significantly less likely to provide translation services; seven of 65 (10.8%) chain pharmacies in our 2006 study regularly provided translated instructions, compared to 61 of 124 (49.2%) independent pharmacies. More than 80% of all pharmacies included in the study lacked systematic approaches for identification and documentation of linguistic needs and for informing patients of translation capabilities (e.g., language preferences indicated in electronic records, multilingual signage alerting customers to available services) [11]. Studies conducted elsewhere in the US report similar gaps in services [12–14].

Recognizing the risks of inadequate comprehension of medication instructions, including poor adherence, errors, and adverse drug events [15], New York lawyers for the Public Interest and Make the Road New York led advocates in New York State (NYS) and NYC in grass-roots campaigns to mandate more systematic and comprehensive approaches to language access services within chain pharmacies. As a result of these campaigns, new regulations were adopted in NYC and NYS between 2008 and 2012 requiring that chain pharmacies provide translation and interpretation services for pharmacy patients speaking the most common non-English languages; however, a system for monitoring compliance by the State Pharmacy Board or other governing body was not included within the regulations. (see Table 5 for a summary of actions and resulting regulations) [16, 17].

The goal of this study is to characterize and assess the impact of language access regulations on services available to LEP patients. In this paper, we focus on capacity to provide written translation and oral interpretation of medication instructions, provision of translated materials, and systematic approaches to identifying language needs among NYC chain pharmacies.

## Methods

This paper reports on surveys of NYC chain pharmacies, conducted in 2006 and 2015, and focused on translation capacity and practices, as well as observations of pharmacy signage conducted in 2015. It is part of a larger project on language services in chain pharmacies being implemented in NYS, New Jersey, and Connecticut, which includes surveys of chain pharmacies; structured observations of pharmacy signage; and surveys and education of LEP community members.

### Data Collection

#### Study Sample

The data used in this analysis come from two random sample telephone surveys of pharmacies: the first conducted in 2006 and the second conducted in 2015. Both surveys used samples selected from current lists of licensed NYS pharmacies maintained by the Office of the Professions, NYS Education Department. Random numbers were generated to select eligible pharmacies. Pharmacies were contacted in sequential order, based on their random number assignment. Trained research assistants conducted all surveys by phone, requesting to speak to a full time, on-duty pharmacist. The research assistants moved to the next pharmacy on the list following a refusal or five unsuccessful attempts to reach a staff pharmacist at the selected pharmacy. Surveys were 5–10 min in length and included responses from pharmacists working during daytime, evening, and weekend shifts.

#### 2015 Survey

Because of the interest in the new laws, the 2015 survey covered chain pharmacies only, including 77 in NYC and 77 in other parts of NYS. Eligibility was based on the definitions of a chain pharmacy used in the regulations, which, in NYC, is four or more locations under common ownership. Because we were examining capacity and practice with respect to translation, we limited our sample to pharmacies located in zip codes with relatively high numbers of residents with limited English proficiency. In NYC, this was defined as pharmacies located in zip codes having at least a 10% foreign born population and a population of 10,000 or more, according to the 2014 American Community Survey.

We contacted 154 NYC chain pharmacies to attain our sample of 77 pharmacies (a 50% response rate). We were unable to reach a pharmacist at 52 pharmacies, 23 pharmacies refused to participate (most commonly citing time constraints), and two pharmacies requested a hard copy of the survey instrument but did not complete it. In addition, two pharmacies asked that we seek permission from a district manager prior to survey completion. These potential respondents were excluded, as outreach to district level management was not part of the study protocol.

#### 2006 Survey

The 2006 survey included 200 NYC pharmacies, 71 of which were chains, according to self-report. From this sample, we identified 48 chain pharmacies that matched eligibility criteria for the 2015 survey (i.e., located in zip codes with a population of at least 10,000, 10% or more of whom were limited English proficient). The response rate for the 2006 survey was 76% [11].

#### Study Variables

The 2006 and 2015 surveys included a set of similar questions, the variation reflecting the desire for greater specificity in the latter survey, as well as sensitivity to the language used by pharmacists, for example “patients” rather than “consumers” (see Appendix 1, Table 6 for relevant questions from the two surveys, according to domain). The surveys focused on (1) pharmacy and pharmacist characteristics and (2) language access capacity and practices that are required by current regulations, specifically:

- Frequency of LEP pharmacy patients;
- Pharmacist characteristics, including gender and birthplace;
- Pharmacy capacity to provide written medication instructions in multiple languages;
- Pharmacy capacity to provide medication counseling in multiple languages, in person or by phone;
- Frequency with which language services (written and oral) are provided to patients with limited English proficiency;
- Practices for notifying patients regarding language services; and
- Practices for identification and documentation of patients needing language services.

The 2015 survey also asked about knowledge of language access laws in NYS and NYC.

#### Observations of Signage

Recognizing the limitations and potential biases in the self-reported data collected through the telephone surveys, we made structured observations of 30 randomly selected pharmacies in three NYC zip codes where at least 10% of the population were LEP Spanish speakers and the population was at least 10,000. Observations focused on adherence to the signage requirements of the language access laws, namely the presence of signs indicating patients’ right to language services, languages included on the signs, location on the pharmacy counter, and sign content.

#### Community Characteristics

Data on LEP prevalence by zip code were obtained from the 2000 Census (2006 pharmacist survey) and the 2014 American Community Survey (2015 pharmacist survey) [18]. These data are available on the US Census website (https://www.census.gov/en.html).

### Analysis

#### Survey Data

Frequency distributions were used to describe the pharmacist and pharmacy characteristics and language translation practices. The differences in pharmacy characteristics and practices between 2006 and 2015 were tested for significance with chi-square tests. Using bivariate and multivariate logistic regression, we examined correlates of practices covered by the regulations: (1) translation of medication instructions, (2) access to phone interpretation, (3) language access signage, and (4) indication of language need in patient records. In the multivariate analysis, we adjusted for survey year; pharmacist gender and birthplace; and percent with limited English proficiency in zip code, dichotomized as 10–20% and greater than 20%. Regression analyses were used to estimate odds ratios and 95% confidence intervals. With the exception of the percentage of residents with limited English proficiency in the pharmacy zip code, all data used in the analyses were derived from survey responses. All variables associated at the *p* < 0.05 in any bivariate analyses were included in the multivariate model.

#### Observation Data

Analysis of observational data focused on proportions with signage at the dispensing counter. Given the small sample size, we did not have significant power for multivariate analyses.

Survey data were analyzed using SAS version 9.3 (Cary, NC). The study protocol was approved by the Institutional Review Board of The New York Academy of Medicine.

## Results

### Sample Characteristics

As shown in Table 1, pharmacists surveyed in 2006 were more likely to be born outside the US and were less likely to report patients with limited English proficiency than were pharmacists surveyed in 2015 (93.8 vs. 100%, *p* < .05).

**Table 1:** Pharmacist and pharmacy characteristics

	Year of survey			
	2006		2015	
	(*N* = 48)		(*N* = 77)	
	*n*	(%)	*n*	(%)
Gender				
Female	31	(64.6%)	46	(59.7%)
Male	17	(35.4%)	31	(40.3%)
Birthplace				
USA	18	(37.5%)	48	(62.3%)*
Outside USA	27	(56.3%)	24	(31.2%)
Missing	3	(6.3%)	5	(6.5%)
Chain size				
4–7 locations	Not available		11	(14.3%)
8 locations or more			66	(85.7%)
Have LEP patients				
Have LEP patients ever	45	(93.8%)	77	(100.0%)*
Have LEP patients daily	41	(85.4%)	69	(89.6%)
LEP patient language				
Spanish	39	(81.3%)	56	(72.7%)
Chinese	9	(18.8%)	18	(23.4%)
Russian	9	(18.8%)	11	(14.5%)
Italian	2	(4.2%)	8	(10.7%)
Another language	6	(12.5%)	21	(27.3%)
Knowledge of language access laws				
Familiarity with language laws	Not applicable		56	(73.7%)
Learned about laws from chain	Not applicable		42	(75.0%)
Learned about laws through State Pharmacy Board	Not applicable		15	(26.8%)
Learned about laws on own	Not applicable		2	(3.6%)
Learned about laws through pharmacist organization	Not applicable		1	(1.8%)
Other	Not applicable		10	(17.9%)

Data source: Authors analysis of 2006 and 2015 random sample phone survey of NYC chain pharmacies

*p < 0.05

### Language Capacity and Practice

Compared to pharmacies surveyed in 2006, those surveyed in 2015 were significantly more likely to report capacity to print translated prescription medication labels (80.4% in 2006 versus 100% in 2015, *p* < 0.001), translating labels daily (15.4 vs. 66.7%, *p* < 0.001), access to a telephone interpretation service (20.8% vs. 90.8%, *p* < 0.001), and use of the telephone service (20.8 vs. 68.8%, *p* < 0.001). In addition, there were reported increases in language needs being part of the patient record (19.4 vs. 49.4%, *p* < 0.01) and written notices or signs indicating access to language services (7.7 vs. 85.7%, *p* < .001). In 2015, just over 75% of pharmacists reported that efforts had been made to hire bilingual staff, compared to 37.5% in 2006 (*p* < .001) (see Table 2). Analysis by chain suggests some variability, with greater compliance to the language access regulations at national chains as compared to local chains and pharmacies located within “big box” stores and supermarkets (see Table 3). Analysis by language showed the greatest translation capacity for Spanish (98.7%), followed by Chinese (75.3%) and Russian and Italian (68.8% each) (data not shown).

**Table 2:** Language access services

	Year of survey			
	2006		2015	
	(*N* = 48)		(*N* = 77)	
	*n*	(%)	*n*	(%)
Translated labels				
Capacity to print translated labels	37	(80.4%)	77	(100.0%)***
Print translated labels daily^a^	6	(15.4%)	44	(66.7%)***
Telephone interpretation service				
Access to telephone interpretation service	10	(20.8%)	69	(90.8%)***
Ever use telephone interpreter service	10	(20.8%)	53	(68.8%)***
Language need identified by^b^				
Staff observe that patient is LEP	13	(41.9%)	61	(79.2%)***
Indicated in patient record	6	(19.4%)	38	(49.4%)**
Patient/family member requests service	18	(58.1%)	33	(42.9%)
Indicated on paper/electronic prescription	3	(9.7%)	17	(22.1%)
Other	7	(22.6%)	7	(9.1%)
Patients aware of language services through^c^				
Written notice or sign	3	(7.7%)	66	(85.7%)***
Pharmacy staff informs them	21	(53.9%)	11	(14.3%)***
Word of mouth	5	(12.8%)	8	(10.4%)
Health provider informs them	0	(0.0%)	6	(7.8%)
Patient needs to ask	4	(10.3%)	2	(2.6%)
Other	3	(7.7%)	4	(5.2%)
Pharmacy made effort to hire bilingual staff				
Effort to hire bilingual staff	15	(37.5%)	53	(75.7%)***

Data source: Authors analysis of 2006 and 2015 random sample phone survey of NYC chain pharmacies

**p < 0.01; ***p < 0.001

^a^Among pharmacies with daily encounters with LEP patients

^b^Among pharmacies that identify language need

^c^Among pharmacies that offer phone or print language services

**Table 3:** Language access services by chain

	National pharmacy chains						Other chains* (*N* = 18)	
	Chain “A”		Chain “B”		Chain “C”			
Language services	*n* = 12	%	*n* = 22	%	*n* = 25	%	*n* = 18	
Capacity to print translated labels	12	(100.0%)	22	(100.0%)	25	(100.0%)	18	(100.0%)
Access to telephone interpretation service	11	(91.7%)	22	(100.0%)	25	(100.0%)	11	(64.7%)***
Written notice or sign re language services	12	(100.0%)	22	(100.0%)	22	(91.7%)	10	(55.6%)***

Data source: Authors analysis of 2006 and 2015 random sample phone survey of NYC chain pharmacies. “Other chains” including a mixture of local chains and pharmacies within supermarkets and “big-box” stores

***p < 0.001

Results from in-person observations conducted in 2015 were relatively consistent with self-report: 80% of observed pharmacies had the required signage at the dispensing counter (data not shown).

In multivariate analyses controlling for pharmacist gender, pharmacist birthplace, and percent LEP in zip code, survey year (2015 compared to 2006) predicted each of the outcomes examined (i.e., requirements of the law): daily translation of medication instructions (AOR 11.7, *p* < 0.001), access to phone interpretation (AOR 69.7, *p* < 0.0001), indication of language need in patient records (AOR 6.8, *p* < 0.001), and language access signage (AOR 131.1, *p* < 0.0001(see Table 4).

**Table 4:** Adjusted odds ratios for provision of language services in pharmacies

Year	Daily translation of labels		Telephone interpretation		LEP in patient record		Written notice of translation	
2006	Reference		Reference		Reference		Reference	
2015	11.7	(3.8, 36.1)***	69.4	(15.9, 303.3)****	6.8	(2.2, 21.0)***	131.1	(23.4, 734.8)****

Data source: Authors analysis of 2006 and 2015 random sample phone survey of NYC chain pharmacies. Odds are adjusted for gender, birthplace (USA or outside USA), and percent LEP in zipcode (10–20%, > 20%)

*p* < ***; *p* < 0.001; ****p < 0.0001

## Discussion

Our findings demonstrate that between 2006 and 2015 there was a significant improvement in the capacity of NYC chain pharmacies to provide language access services, consistent with NYC and NYS regulations adopted between 2008 and 2012 (Table 5). A significantly higher proportion of chain pharmacies surveyed reported being able to print translated medication labels, providing translated labels to LEP patients, access and use of telephone interpreter services, multilingual signage informing patients of language services, and documentation of language needs in patient records—all of which are requirements of the new regulations and laws adopted in New York. These findings illustrate the potential impact of grass-roots advocacy and resultant policy change on institutional practices that impact significant portions of the population.

**Table 5:** Summary of New York State and New York City Pharmacy Language Access Requirements, According to Jurisdiction

Areas covered in legislation/settlements	NYS Attorney General agreements	NYC Language Access in Pharmacies Act	NYS SafeRx legislation
Effective years	• 2008–2013	• 2010 and on	• 2013 and on
Covered pharmacies	• Seven chain pharmacies identified in the legal complaint	• Chains with 4+ stores in NYC	• Chains with 8+ stores in NYS and mail orders
Signage requirements	• Pharmacy Patients’ Bill of Rights on websites	• Sign indicating rights to language assistance services in a conspicuous location near the pharmacy counter	• Sign indicating rights to language assistance services in a conspicuous location near the pharmacy counter
	• Notices in each pharmacy on rights to language assistance services		
Interpretation (oral)	• Interpretation services required, specifically remote telephone interpretation	• Interpretation services required: pharmacies may choose methods, including use of competent, bilingual staff	• Interpretation services required: pharmacies may choose methods, including use of competent, bilingual staff
Translation (written)	• All prescription labels, warning labels and vital documents • Languages: Spanish, Chinese, Italian, Russian, French; five additional based on languages spoken by customers	• All prescription labels, warning labels and vital documents • Languages: top 7 languages spoken in NYC	• All prescription labels, warning labels and vital documents • Languages: those spoken by 1% or more of the population by region, not exceeding 7 per region.
Staff training	• Pharmacy staff to be trained on language access policy & equipment	• No training requirements	• No training requirements
Enforcement/penalties	• Record-keeping to enable monitoring by NYS Attorney General • Patient complaint system	• Violations can incur fines	• No new fines or penalties

Our ability to conduct surveys using similar protocols before and after the implementation of pertinent regulations and laws is a particular strength of this study. The significant change in chain pharmacy practices related to language access over just a few years is notable, given early concerns related to feasibility and liability exposure, which were expressed by pharmacists when we conducted our earlier study [11].

As far as we know, New York’s pharmacy language regulations are unique. The findings reported here could thus be useful in the development of similar policy efforts in other jurisdictions. Most of the pharmacies included in this study are part of multistate chains that have developed systems for the provision of language services. Promoting and utilizing those systems outside of New York, seemingly, could be accomplished with relative ease.

This study has several limitations. At both points in time, data were largely self-reported, and were therefore subject to recall errors and social-desirability bias. Although the reliability of recall should be similar for the 2006 and 2015 surveys, it is possible that, given the change in regulations, social-desirability bias may have been stronger in 2015. In addition, the pharmacist perspective represents just a part of the story: receipt of language-appropriate instructions by patients with limited English proficiency, improved comprehension, reduced medication errors, and better health are the ultimate goals. We have been surveying Spanish-speaking LEP patients as part of the larger study, to better understand their experience with language services in chain pharmacies and the implications thereof. Finally, similar research on chain pharmacies in other states is necessary to verify that the changes reported here were the result of New York regulations, rather than more general shifts in pharmacy practice. We are carrying out surveys and structured observations in communities in Connecticut and New Jersey (contiguous states to NYS) with high numbers of LEP residents to address this concern.

Despite these limitations, the significant changes documented in this study were largely consistent across the specific language services covered by the regulations. The results make a convincing case for the effectiveness of the laws and regulations implemented and for their adaptation in other states and municipalities.

## Appendix 1

**Table 6:** Principal domains and questions for comparison of 2006 and 2015 surveys.

Domain	2006 Survey	2015 Survey
Limited English proficient patients at pharmacy	• Do you have clients who speak: Spanish/Chinese/Russian/[other languages]? • If yes [per language], daily/not daily?	• How often does your pharmacy have patients who primarily speak: Spanish/Chinese/Russian/Italian/Other?
Label translation	• Do you have the capacity to print translated medication labels and/or medication leaflets (package inserts) for customers? • How often do you personally provide translated labels at this pharmacy	• Does your pharmacy have the capacity to print translated medication labels and other translated materials for patients? • Thinking again about the last 3 months, how often would you say that translated labels were provided at your pharmacy?
Telephone interpretation	• Do you have access to telephone translation for medication counseling? • How often do you use the telephone translation service?	• Do you have access to telephone interpretation for medication counseling? • Thinking about the last three months, how often have you used the telephone interpretation service?
Identification of patient language needs	• In general, how do you determine that someone should get translated information?	• In general, how would pharmacy staff know that someone needs language services?
Patients awareness of language services	• How would customers know that translated information is available?	• How would patients know that language services are available?
Effort to hire bilingual staff	• Have specific efforts been made in your pharmacy to hire staff who speak languages that are commonly used in your community?	• Have specific efforts been made in your pharmacy to hire staff who speak languages that are commonly used in your community?
